# 老年多发性骨髓瘤患者接受自体造血干细胞移植的疗效和安全性：单中心回顾性研究

**DOI:** 10.3760/cma.j.issn.0253-2727.2022.02.009

**Published:** 2022-02

**Authors:** 蓓晖 黄, 娟 李, 外一 邹, 俊茹 刘, 景立 谷, 晓哲 李, 美兰 陈, 丽芬 邝

**Affiliations:** 中山大学附属第一医院，广州 510080 First Affiliated Hospital, Sun Yat-sen University, Guangzhou 510080, China

**Keywords:** 多发性骨髓瘤, 老年, 自体造血干细胞移植, 治疗效果, 毒副作用, Multiple myeloma, Elderly, Autologous transplantation, Treatment efficacy, Toxicity

## Abstract

**目的:**

评估65岁以上老年多发性骨髓瘤（MM）患者接受自体造血干细胞移植的疗效和安全性。

**方法:**

回顾性分析自2006年6月1日至2020年7月31日在中山大学附属第一医院诊断并接受含新药诱导方案序贯自体造血干细胞移植的22例65岁以上MM患者的疗效和安全性。接受移植的老年患者均在移植前对重要脏器功能进行评估，自2016年起同时采用国际骨髓瘤工作组虚弱评分评估移植适应证。

**结果:**

22例患者接受移植时的中位年龄为66.75（IQR 4.50）岁。共20例患者接受了造血干细胞动员，中位采集造血干细胞单个核细胞数为4.53×10^8^/kg，CD34^+^细胞数为3.37×10^6^/kg，中位采集次数2次。造血干细胞回输后中性粒细胞植入中位时间11 d，血小板植入中位时间13 d，移植后100 d治疗相关死亡率为0。中位随访48.7个月，中位无进展生存时间未达到，中位总生存时间为111.8个月。

**结论:**

对于经过筛选的≥65岁老年MM患者，自体造血干细胞移植是安全有效的治疗方案。

## Abstract (en)

**Objective:**

To evaluate the efficacy and safety of autologous hematopoietic stem cell transplantation (auto-HSCT) in elderly patients (≥65 years old) with multiple myeloma (MM).

**Methods:**

From June 1, 2006 to July 31, 2020, 22 MM patients (≥65 years old) who were diagnosed in the First Affiliated Hospital, Sun Yat-sen University and received novel drug induction followed by auto-HSCT were analyzed retrospectively. These patients were evaluated for important organ functions before transplantation, and the International Myeloma Working Group frail score was used in 2016 to screen out transplant-eligible patients.

**Results:**

The median (interquartile range, IQR) age at the time of transplantation of the 22 patients was 66.75 (IQR 4.50) years. A total of 20 patients received stem cell mobilization. The median number of mononuclear cells collected was 4.53×10^8^/kg, that of CD34^+^ cells was 3.37×10^6^/kg, and the median number of apheresis procedures performed was 2. After stem cell transfusion, the median time of neutrophil implantation was 11 days, that of platelet implantation was 13 days, and the treatment-related mortality was 0 at 100 days after transplantation. The median follow-up was 48.7 months. The median time to progression time was not reached, and the median overall survival time was 111.8 months.

**Conclusion:**

Auto-HSCT is a safe and effective treatment for selected elderly patients of 65 years or older with MM.

多发性骨髓瘤（multiple myeloma，MM）是一种老年性疾病[1]。美国梅奥诊所的一项研究证实，与20世纪50年代相比，21世纪初的老年人群中确诊MM的病例数显著增加，确诊MM的中位年龄从70岁增加到74岁，80岁及以上新诊断患者的比例增加了一倍[2]。如何更好地为老年MM患者选择合适的方案非常重要。在过去的20年里，大量MM新药上市，极大地改善了MM患者的疗效和生存，使MM的治疗逐渐转变为慢性病的治疗。但即便如此，大量临床研究数据表明，自体造血干细胞移植（auto-HSCT）目前仍然是65岁以下MM患者的首选治疗方案[1],[3]。但65岁以上患者的数据较少，主要原因为大多数随机对照临床研究为了减少退出率和严重不良反应的发生，纳入的研究对象均为65岁以下患者，但这并不意味着auto-HSCT在老年患者中不可行[3]–[4]。一些回顾性研究提示对于适合移植的65岁以上MM患者，接受auto-HSCT的患者生存时间优于未接受移植的患者，与年轻患者的疗效和无进展生存时间无差异。但目前关于适合行auto-HSCT患者的年龄选择国内外尚无明确共识，大多数指南推荐经严格筛选的65岁以上的MM患者可考虑接受auto-HSCT。

美国的初治MM患者中约30％接受了auto-HSCT，但我国MM患者接受auto-HSCT比例明显低于欧美国家，这与我国许多医师和患者对auto-HSCT认识不足相关。在此前提下，老年患者接受auto-HSCT的比例更低。国内年龄≥65岁MM患者接受auto-HSCT的报道罕见。本中心自2006年起对适合移植的患者采用新药诱导序贯auto-HSCT序贯维持治疗的整体治疗模式，截至2020年7月31日，治疗了273例患者，其中22例患者为65岁及以上，疗效理想，患者耐受性好，现将本中心数据总结如下。

## 病例与方法

1. 病例：回顾性分析自2006年6月1日至2020年7月31日在我院诊断并接受含新药诱导方案序贯auto-HSCT且移植时年龄≥65岁MM患者的基线特征、疗效和安全性。其中高危细胞遗传学定义为荧光原位杂交检测到17p−、t（4;14）、t（14;16）中的一个或多个。所有拟行auto-HSCT的患者在移植前均接受了器官功能评估，要求重要脏器无严重受损，具体如下：①肝功能检查转氨酶不超过正常上限的3倍，胆红素不超过正常上限的1.5倍；②左心室射血分数>40％，心功能 NYHA分级1～2级；③无重大肺部疾病，若可疑慢性肺部疾病，需完善肺功能评估，一秒用力呼气容积占预计值百分比≥60％和（或）弥散功能占预计值百分比 ≥60％。自2016年起，在诱导治疗前后均用国际骨髓瘤工作组（IMWG）虚弱评分的3个量表［日常生活能力（activity of daily living, ADL）、日常生活工具能力（instrumental activity of daily living, IADL）和Charlson指数］对所有患者进行评分，对于诱导治疗后评分为体能状态良好（Fit）的患者可以选择移植，对于评分为体能状态一般（Intermediate）的患者则通过与患者及家属充分沟通后再决定是否移植，对于评分为体能状态差（Unfit）的患者不选择移植。

2. 治疗方案：诱导化疗方案包括VD方案（硼替佐米 1.3 mg·m^−2^·d^−1^，静脉推注，第1、4、8、11天；地塞米松 20 mg/d，静脉滴注，第1～4天；21 d为1个疗程）、PAD方案（硼替佐米1.3 mg·m^−2^·d^−1^，静脉推注，第1、4、8、11天；脂质体阿霉素40 mg·m^−2^·d^−1^，静脉滴注，第4天；地塞米松 20 mg/d，静脉滴注，第1～4天；28 d为1个疗程）、VDDT方案（硼替佐米1.3 mg·m^−2^·d^−1^，静脉推注，第1、4、15、18天；脂质体阿霉素40 mg·m^−2^·d^−1^，静脉滴注，第4、18天；地塞米松20 mg/d，静脉滴注，第1～4、15～18天；沙利度胺200 mg/d，每晚口服；28 d为1个疗程）、RAD方案（来那度胺25 mg/d，口服，第1～21天；脂质体阿霉素40 mg·m^−2^·d^−1^，静脉滴注，第4天；地塞米松20 mg/d，静脉滴注，第1～4天；28 d为1个疗程）、VRD方案（硼替佐米1.3 mg·m^−2^·d^−1^，静脉推注，第1、4、8、11天；来那度胺25 mg/d，口服，第1～21天；地塞米松 20 mg/d，静脉滴注，第1～4天；28 d为1个疗程）。

采用大剂量环磷酰胺（CTX）+粒细胞集落刺激因子（G-CSF）方案（CTX 3.0 g·m^−2^·d^−1^，静脉滴注，第1天；G-CSF 300 µg/d，皮下注射，第2天至造血干细胞采集）或单用G-CSF（G-CSF 300 µg/d，皮下注射，连续5 d）动员。

预处理方案采用大剂量美法仑（HD-MEL）或CVB方案。美法仑根据肌酐清除率进行调整：肌酐清除率>50 ml/min时，美法仑100 mg·m^−2^·d^−1^，静脉滴注，−3～−2 d；肌酐清除率为30～50 ml/min时，美法仑剂量减低至70 mg·m^−2^·d^−1^，静脉滴注，−3～−2 d；肌酐清除率<30 ml/min时，美法仑剂量降低至50 mg·m^−2^·d^−1^，静脉滴注，−3～−2 d。CVB方案：白消安0.8 mg·kg^−1^·（6 h）^−1^，静脉推注，−8～−6 d；依托泊苷8～10 mg·kg^−1^·d^−1^，静脉滴注，−5～−4 d；CTX 40～50 mg·kg^−1^·d^−1^，静脉滴注，−3～−2 d。中性粒细胞植入的定义是连续3 d中性粒细胞绝对计数≥0.5×10^9^/L，血小板植入的定义是连续7 d PLT≥20×10^9^/L。治疗相关死亡率（TRM）定义为auto-HSCT后100 d的死亡率。为评估移植方案的安全性和不良反应，根据国家癌症研究所通用术语标准4.0版对不良事件进行描述。

20例患者采用外周血造血干细胞移植，2例患者采用骨髓移植，其中1例因早年无冻存液无法冻存外周血干细胞，1例因肾功能不全未采用CTX动员而改为骨髓移植。

移植后常规进行维持治疗，维持治疗方案包括沙利度胺（15例，68.2％）、干扰素（1例，4.5％）、沙利度胺+干扰素（3例，13.3％）、来那度胺（3例，13.3％）。

3. 随访：通过住院、门诊病历和电话随访。所有患者随访至2021年2月28日，中位随访时间48.7（IQR 60.6）个月。

4. 疗效评价：疗效评价参照IMWG 2006疗效评价标准[5]，分为完全缓解（CR）、非常好的部分缓解（VGPR）、部分缓解（PR）、疾病稳定（SD）以及疾病进展（PD）。总有效率（ORR）为CR率、VGPR率、PR率之和。至疾病进展时间（TTP）定义为从诱导治疗开始到疾病进展的时间，总生存（OS）时间定义为从诱导治疗开始到死亡或随访截止的时间。

5. 统计学处理：采用SPSS 23.0软件进行统计学分析。计量资料采用*M*（IQR）或*M*（范围）描述，分类变量采用例数（百分比）描述。计量资料的比较采用*t*检验或非参数检验，分类变量采用Pearson *χ*^2^检验。TTP和OS的分析采用Kaplan-Meier法，并采用Log-rank法比较生存曲线。*P*<0.05为差异有统计学意义。

## 结果

1. 临床特征：自2006年6月1日至2020年7月31日，有273例MM患者在中山大学附属第一医院接受了auto-HSCT，其中22例（8.1％）移植时年龄≥65岁，251例（91.9％）移植时年龄<65岁。22例接受移植的老年患者中男16例（72.7％），女6例（27.3％）。M蛋白类型方面，IgG型13例（59.1％），IgA型5例（22.7％），IgD型1例（4.5％），轻链型3例（13.6％）。ISS分期方面，Ⅰ期5例（22.7％），Ⅱ期13例（59.1％），Ⅲ期4例（18.2％）。3例（14.3％）患者骨髓浆细胞比例>60％，14例（66.7％）浆细胞比例10％～60％，4例（19.0％）浆细胞比例<10％。实验室检查方面，3例（13.6％）起病时肌酐≥177 µmol/L，12例（54.5％）HGB<100 g/L，4例（18.2％）有高钙血症（校正钙≥2.75 µmol/L），3例（15.8％）乳酸脱氢酶升高。

这些患者发病的中位年龄66（IQR 7）岁，接受移植时的中位年龄为66.75（IQR 4.50）岁。15例患者在诊断时进行了荧光原位杂交，其中5例（33.3％）有高危细胞遗传学改变。其中骨髓浆细胞比例小于10％的患者均通过多部位骨髓穿刺证实多部位有克隆性浆细胞，且证实器官损伤与骨髓瘤相关，和（或）病理活检证实有浆细胞瘤。

2016年后有11例年龄超过65岁的患者接受auto-HSCT，其中8例在诱导前IMWG虚弱评分为Fit，1例为Intermediate，2例为Unfit。诱导治疗后再次评估，2例Unfit患者中1例转变为Fit，1例转变为Intermediate；1例Intermediate转变为Fit。

2. 治疗情况及疗效：所有患者接受了2至6个周期的诱导治疗，19例（86.4％）患者接受了以硼替佐米为基础的诱导方案治疗，其中7例接受了VD方案，12例接受了PAD方案；2例（9.1％）患者接受了来那度胺方案诱导治疗，均为RAD方案；1例（4.5％）患者接受了以硼替佐米和来那度胺为基础的诱导方案治疗，包括2个疗程VCD方案和4个疗程VRD方案。诱导治疗后的ORR为95.5％，其中CR 10例（45.5％），VGPR 7例（31.8％），PR 4例（18.2％）。22例患者中有1例维持治疗后失访，其余21例患者维持治疗后（移植后3个月评估疗效）的ORR为90.5％，其中CR 13例（61.9％），VGPR 5例（23.8％），PR 1例（4.8％）。

3. 造血干细胞动员情况：共20例患者接受了造血干细胞动员，其中18例接受大剂量CTX+G-CSF（1例第一次动员采集造血干细胞不足，后单用G-CSF再次动员），2例接受了单用G-CSF动员。20例患者中位采集造血干细胞单个核细胞数为4.53（IQR 2.99）×10^8^/kg，CD34^+^细胞数为3.37（IQR 2.50）×10^6^/kg，中位采集次数2（IQR 1）次，动员期间的住院时间为12（IQR 1.75）d，粒细胞缺乏时间为3（IQR 1.75）d，7例（63.4％）患者发生3～4级血小板减少。

4. 预处理及移植情况：22例患者中有20例为早期移植，即确诊后即按移植方案治疗，且在造血干细胞动员后尽快行auto-HSCT，这些患者从诊断到进行auto-HSCT的中位时间为7.2（IQR 2.0）个月。2例患者为晚期移植，指患者在复发难治后才选择移植，2例患者从诊断到移植的时间分别为49.6个月和53.2个月。

11例（50.0％）患者接受大剂量美法仑预处理方案，其中9例（40.9％）患者接受美法仑总量200 mg/m^2^，另外各有1例（4.5％）患者接受美法仑总量140 mg/m^2^和100 mg/m^2^。11例（50.0％）患者接受CVB预处理方案。

造血干细胞回输后中性粒细胞植入的中位时间为11（IQR 3）d，中位血小板植入时间为13（IQR 12）d，移植后100 d TRM为0。

5. 生存情况：随访截至2021年2月28日，中位随访48.7（IQR 60.6）个月，9例（40.9％）患者复发或疾病进展，中位TTP未达到，11例（50％）患者死亡，中位OS时间为111.8（36.2～185.2）个月。

## 讨论

尽管目前有大量新药被开发并用于MM治疗，但auto-HSCT在适合移植患者中的治疗地位仍然无法替代。目前所有指南均推荐，对于适合移植的65岁以下患者，首选新药诱导序贯auto-HSCT。但对于年龄超过65岁的患者而言，是否积极采用移植治疗仍有一定争议[3]。大部分欧美国家均推荐auto-HSCT的适应证可延长至70岁，部分指南甚至推荐可延长至80岁。欧洲骨髓移植（EBMT）登记数据显示，近年来，65岁以上接受auto-HSCT的患者数量有所增加，2006–2010年间65～69岁接受auto-HSCT的患者从2001–2005年的2 478例（占移植患者的14.1％）上升至3 860例（占移植患者的15.8％）[6]。

老年患者行auto-HSCT最关注的问题是是否可以与年轻患者获得同样的疗效以及是否存在严重的治疗并发症及TRM。早在传统化疗时代，研究者就开始关注老年患者是否可以耐受auto-HSCT[7]–[8]。一些临床研究结果显示如果患者体能状态良好且无严重合并症，患者的年龄上限可以放宽至75岁。在新药时代，多个临床研究和真实世界研究也提示经过筛选的老年移植患者的生存优于不接受移植者或与年轻患者相当[9]–[12]。本研究组的研究结果也显示，对于经过筛选的老年患者，auto-HSCT疗效及患者的生存较理想。

老年患者接受auto-HSCT另一个需要密切关注之处是不良反应及治疗耐受性，因此如何挑选适合移植的患者至关重要，其中体能状态评分对于老年患者是否可以耐受移植十分重要。多个研究认为，体能状态评分为Unfit的患者不合适行auto-HSCT。但是值得注意的是，部分老年患者起病时体能状态较差，因此需要在诱导治疗后对这些患者再次进行体能评估，这时患者的评分才能真正反映患者的体能状态，从而评估患者是否可以耐受移植。本研究患者中，有2例在起病时体能状态评估为Unfit，但经过诱导治疗后1例转变为Fit，1例转变为Intermediate，反映了诱导治疗后再次评估体能状态的重要性。本研究中，老年患者采用大剂量CTX+G-CSF方案动员造血干细胞后的粒细胞缺乏持续时间、血小板减少程度均在可控范围；预处理期间的粒细胞和血小板植入时间也与文献报道的年轻患者无明显差异，TRM为0。上述结果提示，对于精确筛选的老年MM患者，auto-HSCT是安全有效的治疗方案[13]。

老年患者auto-HSCT的另一个关注点是采集的造血干细胞数量是否足够。既往研究提示高龄是影响造血干细胞采集的危险因素之一，会导致植入不良，甚至影响维持治疗的进行[11]。Stettler等[12]发现，70岁以上的老年患者造血干细胞动员数显著减少（*P*＝0.010），导致造血干细胞采集次数增加、血小板减少持续时间显著延长，住院时间显著延长。但我们的研究中，20例接受造血干细胞动员的患者中仅1例患者第一次动员失败，且经过再次动员后可以采集足够数量的造血干细胞。原因一方面可能是患者年龄均小于70岁，另一方面，患者均经过严格评估才进行移植，提示对于老年患者，需要更加严格地筛选。

老年患者auto-HSCT预处理方案是否需要减量也有一定的争议。在传统化疗时代，一项前瞻性研究分析了年龄≥70岁患者行auto-HSCT的可行性和有效性，结果发现接受美法仑200 mg/m^2^（MEL-200）预处理的患者TRM过高（16％），因此将剂量降低至140 mg/m^2^（MEL-140），并发现MEL-140在老年患者中毒性较小，且与MEL-200相比疗效相同[14]。另一项研究发现老年患者的移植不良反应与年轻患者相当，但老年患者有61.5％采用美法仑140 mg/m^2^预处理方案[13]。在英国骨髓瘤Ⅺ试验中，采用美法仑140 mg/m^2^和美法仑200 mg/m^2^的老年患者的预后相当[9]。EBMT注册数据库的研究报告显示，在总体人群中，美法仑140 mg/m^2^和200 mg/m^2^的预后无显著差异，但疗效不佳的患者可以从美法仑200 mg/m^2^中获益[15]。而本研究中，我们并未根据患者的年龄减量，而是通过肾功能决定是否减量，这种减量原则是安全有效的。总体而言，目前老年患者auto-HSCT的预处理方案无明确的剂量推荐，但对于肾功能不全、一般情况较差的患者，可考虑美法仑剂量减低至140 mg/m^2^。

## References

[1] Bergin K, McQuilten Z, Moore E (2017). Myeloma in the Real World: What Is Really Happening?[J]. Clin Lymphoma Myeloma Leuk.

[2] Turesson I, Velez R, Kristinsson SY (2010). Patterns of multiple myeloma during the past 5 decades: stable incidence rates for all age groups in the population but rapidly changing age distribution in the clinic[J]. Mayo Clin Proc.

[3] Al Hamed R, Bazarbachi AH, Malard F (2019). Current status of autologous stem cell transplantation for multiple myeloma[J]. Blood Cancer J.

[4] Wildes TM, Rosko A, Tuchman SA (2014). Multiple myeloma in the older adult: better prospects, more challenges[J]. J Clin Oncol.

[5] Durie BG, Harousseau JL, Miguel JS (2006). International uniform response criteria for multiple myeloma[J]. Leukemia.

[6] Auner HW, Szydlo R, Hoek J (2015). Trends in autologous hematopoietic cell transplantation for multiple myeloma in Europe: increased use and improved outcomes in elderly patients in recent years[J]. Bone Marrow Transplant.

[7] Siegel DS, Desikan KR, Mehta J (1999). Age is not a prognostic variable with autotransplants for multiple myeloma[J]. Blood.

[8] Facon T, Mary JY, Hulin C (2007). Melphalan and prednisone plus thalidomide versus melphalan and prednisone alone or reduced-intensity autologous stem cell transplantation in elderly patients with multiple myeloma (IFM 99-06): a randomised trial[J]. Lancet.

[9] Pawlyn C, Cairns D, Menzies T (2022). Autologous stem cell transplantation is safe and effective for fit older myeloma patients: exploratory results from the Myeloma XI trial[J]. Haematologica.

[10] Lemieux C, Muffly LS, Rezvani A (2021). Outcomes with autologous stem cell transplant vs. non-transplant therapy in patients 70 years and older with multiple myeloma[J]. Bone Marrow Transplant.

[11] Muchtar E, Dingli D, Kumar S (2016). Autologous stem cell transplant for multiple myeloma patients 70 years or older[J]. Bone Marrow Transplant.

[12] Stettler J, Novak U, Baerlocher GM (2017). Autologous stem cell transplantation in elderly patients with multiple myeloma: evaluation of its safety and efficacy[J]. Leuk Lymphoma.

[13] Wiebach H, Gezer D, Brummendorf TH (2020). Tolerability of high dose chemotherapy and autologous stem cell transplantation in elderly patients with multiple myeloma: A single-center retrospective analysis[J]. Curr Res Transl Med.

[14] Badros A, Barlogie B, Siegel E (2001). Autologous stem cell transplantation in elderly multiple myeloma patients over the age of 70 years[J]. Br J Haematol.

[15] Auner HW, Iacobelli S, Sbianchi G (2018). Melphalan 140 mg/m^2^ or 200 mg/m^2^ for autologous transplantation in myeloma: results from the Collaboration to Collect Autologous Transplant Outcomes in Lymphoma and Myeloma (CALM) study. A report by the EBMT Chronic Malignancies Working Party[J]. Haematologica.

